# Osteomyelitis in Immunocompromised children and neonates, a case series

**DOI:** 10.1186/s12887-021-03031-1

**Published:** 2021-12-11

**Authors:** Bryan Foong, Kenneth Pak Leung Wong, Carolin Joseph Jeyanthi, Jiahui Li, Kevin Boon Leong Lim, Natalie Woon Hui Tan

**Affiliations:** 1 Singapore General Hospital, Singapore, Singapore; 2 Department of Orthopedic Surgery, KK Women’s and Children’s Hospital, Singapore, Singapore; 3 Yong Loo Lin School of Medicine, National University of Singapore, Singapore, Singapore; 4 Duke-National University of Singapore Medical School, Singapore, Singapore; 5 Deparment of Pediatrics, KK Women’s and Children’s Hospital, Singapore, Singapore; 6 Infectious Disease Service, Department of Pediatrics, KK Women’s and Children’s Hospital, Singapore, Singapore; 7 Lee Kong Chian School of Medicine, Nanyang Technological University, Singapore, Singapore

**Keywords:** Osteomyelitis, Immunocompromised children, Neonates

## Abstract

**Background:**

Osteomyelitis in immunocompromised children can present differently from immunocompetent children and can cause devastating sequelae if treated inadequately. We aim to review the aetiology, clinical profile, treatment and outcomes of immunocompromised children with osteomyelitis.

**Methods:**

Retrospective review of all immunocompromised children aged < 16 years and neonates admitted with osteomyelitis in our hospital between January 2000 and January 2017, and referred to the Paediatric Infectious Disease Service.

**Results:**

Fourteen patients were identified. There were 10 boys (71%), and the median age at admission was 70.5 months (inter-quartile range: 12.3–135.0 months). Causal organisms included, two were *Staphylococcus aureus*, two were *Mycobacterium bovis* (BCG), and one each was *Mycobacterium tuberculosis*, *Pseudomonas aeruginosa*, *Stenotrophomonas maltophilia*, *Burkholderia pseudomallei* and *Rhizopus* sp. One patient had both *Clostridium tertium* and *Clostridium difficile* isolated. Treatment involved appropriate antimicrobials for a duration ranging from 6 weeks to 1 year, and surgery in 11 patients (79%). Wherever possible, the patients received treatment for their underlying immunodeficiency. For outcomes, only three patients (21%) recovered completely. Five patients (36%) had poor bone growth, one patient had recurrent discharge from the bone and one patient had palliative care for underlying osteosarcoma.

**Conclusions:**

Although uncommon, osteomyelitis in immunocompromised children and neonates can be caused by unusual pathogens, and can occur with devastating effects. Treatment involves prolonged administration of antibiotics and surgery. Immune recovery also seems to be an important factor in bone healing.

## Background

An immunocompromised state describes individuals with an impairment of the immune system which can arise from primary or secondary immunodeficiencies [1, 2]. OM is the infection of bone by haematogenous, direct or contiguous invasion. Risk factors for OM in children include immunodeficiency, sickle cell disease, trauma and presence of indwelling foreign bodies such as arterial lines [3]. *Staphylococcus aureus* is the most common causal pathogen in OM. Other typical organisms include *Streptococcus pyogenes* and *Streptococcus pneumoniae* [4, 5]. Atypical pathogens such as Gram negative bacteria, atypical mycobacterium and fungi are more commonly seen in immunocompromised children [6–8].

An immunocompromised state can influence the presentation, diagnosis and management of osteomyelitis (OM). A child with OM typically presents with fever, bone pain, reduced ambulation, bone swelling and generalised malaise [4, 5]. The infection usually involves the metaphyses of long bones [5].

Raised C-reactive protein (CRP) is a sensitive test for the diagnosis and monitoring of the disease [5, 9]. Plain radiographs are less sensitive in detecting OM as osteolytic lesions only become visible after two to 3 weeks [5]. Increasingly, magnetic resonance imaging (MRI) is becoming the main imaging modality due to the potential to diagnose early and difficult cases, allowing for vital early treatment [10, 11]. Blood and bone cultures are also useful [12].

OM is initially treated empirically based on the suspected organism to avoid further damage to the bone [13]. Once specific sensitivities have been established, the type of antimicrobial is adjusted appropriately. The usual course of antimicrobials is 20 days with a short initial intravenous (IV) phase and a longer oral phase [14]. The role of surgery in children with acute OM is uncertain. It is accepted that surgical drainage under general anaesthesia is crucial if the patient is unresponsive to medical treatment or if there is evidence of an abscess or sequestrum [2, 6, 9].

OM in immunocompromised children and neonates is rare. However, it can cause devastating sequelae, such as pathological fractures, growth disturbances and deformity, warranting quick and aggressive treatment. Due to the limited literature on OM in immunocompromised children and neonates, we aim to review the clinical course of the disease including the initial presentation, diagnostic process, management approach and eventual outcomes of these patients.

## Methods

This is a retrospective case series. The patients were identified from an inpatient registry from our Pediatric Infectious Disease Service. We selected all neonates and children aged < 16 years who had a formal diagnosis of a primary or secondary immunodeficiency admitted for OM between January 2000 and January 2017 in our hospital and referred to the Pediatric Infectious Disease Service. We excluded patients who did not have a confirmed diagnosis of immunodeficiency as well as those who did not have prior treatment or antibiotics. Diagnosis was made based on clinical suspicion as well as blood/tissue cultures. We extracted data concerning patient’s demographics, imaging, microbiology, sensitivities, antimicrobials and outcomes. The study was approved by the Singhealth Centralised Institutional Review Board (CIRB).

## Results

There were 14 patients identified, of which 12 were immunocompromised children, and two were neonates with immature immune systems. There were ten boys (71%) and four girls. The median age at admission was 70.5 months (inter-quartile range: 12.3–135.0 months). Of the 12 immunocompromised children, six of them (50%) had chemotherapy for underlying cancer, two had suspected Mendelian susceptibility to mycobacterial disease (MSMD), and one each had Bruton’s agammaglobulinaemia, familial haemophagocytic lymphohistiocytosis (HLH), diabetes mellitus and bone marrow transplant for underlying Fanconi anemia.

The patient demographics are summarised in Table 1.

**Table 1:** Patient demographics

Patient	Sex	Underlying Condition
1	Female	Acute Lymphoblastic Leukaemia on chemotherapy
2	Male	Bone marrow transplant for underlying Fanconi Anaemia
3	Female	Neuroblastoma on chemotherapy
4	Female	Osteosarcoma on chemotherapy
5	Male	Acute Lymphoblastic Leukaemia on chemotherapy
6	Male	Acute Lymphoblastic Leukaemia on chemotherapy
7	Male	Neonate, born premature at 28 + 5 weeks, now 36 + 6 weeks
8	Male	Neonate, born at term
9	Male	Familial Haemophagocytic Lymphohistiocytosis
10	Male	Suspected Mendelian Susceptibility to Mycobacterial Disease
11	Male	Acute Lymphoblastic Leukaemia on chemotherapy
12	Female	Type 2 Diabetes Mellitus
13	Male	Bruton’s Agammaglobulinaemia
14	Male	Suspected Mendelian Susceptibility to Mycobacterial Disease

As seen in Table 2, our patients presented with one or more of the classical features of acute OM including fever, bone pain, bone swelling and reduced active mobility. Of the four symptoms, the most common feature was bone swelling, seen in 13 patients (92.9%). On the other hand, the least common symptom was limited mobility, seen in only four patients (28.6%). On the whole, fever, bone pain and reduced active mobility were not highly sensitive in detecting OM and had sensitivities of 64.3, 42.9 and 28.6% respectively. Only one patient (7.1%) displayed all four of the classical features. The clinical sign of bone swelling and a raised CRP was more sensitive in detecting OM with sensitivity of 92.9 and 85.7% respectively.

**Table 2:** Clinical presentation of the patients

Patient	Fever (Highest Temperature)	Bone Pain	Bone Swelling^a^	Reduced Active Mobility	Highest CRP (mg/L)	Raised CRP
1	Yes (39.1 °C)	Yes	Yes	Yes	286	Yes
2	Yes (38.7 °C)	Yes	Yes	No	175	Yes
3	Yes (39.7 °C)	Yes	Yes	No	119.7	Yes
4	Yes (39.4 °C)	Yes	No	Yes	89.1	Yes
5	No	No	Yes	No	68.5	Yes
6	Yes (38.8 °C)	No	Yes	No	61.7	Yes
7	No	NA	Yes	No	130	Yes
8	Yes (39.3 °C)	NA	Yes	No	57.1	Yes
9	No	Yes	Yes	No	69.4	Yes
10	Yes (38.2 °C)	NA	Yes	Yes	7.1	No
11	Yes (40.0 °C)	No	Yes	No	Not done	Not done
12	Yes (40.0 °C)	Yes	Yes	No	345.6	Yes
13	No	NA	Yes	Yes	130.8	Yes
14	No	NA	Yes	No	24.8	Yes
% Positive	64.3	66.7	92.9	28.6		92.3

^a^Finding on clinical examination

The sites of infection and isolated pathogens vary widely, as seen in Table 3. Commonly affected sites such as the humerus, femur and tibia were involved in nine patients (64.2%). However, uncommon sites such as flat bones like the base of skull and short bones like the tarsal bones and talus were also involved.

**Table 3:** Summary of patients’ site of infection, pathogen isolated, treatment regimen, surgery and outcome

Patient	Site of Infection	Pathogen Isolated	Empirical Treatment: Antibiotic (days)	Targeted Antimicrobial Treatment: Antibiotic (days)	Surgical Treatment (number of surgeries)	Outcome
1	Proximal Tibia	*Clostridium tertium* and *Clostridium difficile*	IV Cloxacillin (5) IV Tazocin (5)	IV Vancomycin (6) IV Crystalline Penicillin (90) IV Metronidazole (90) Oral Metronidazole (180)	Incision and drainage/ Curettage (5)	Recurrent discharge
2	Distal Tibia	*Stenotrophomonas maltophilia*	IV Meropenem (3)	IV Polymyxin (84) IV Ticarcillin (160) Oral Moxifloxacin (180) Oral Minocycline (184)	Incision and drainage/ Curettage (5)	*Complete resolution
3	Distal Femur	Unable to discern	IV Cloxacillin (5) IV Clindamycin (5) IV Ceftazidime (6)	Oral Ciprofloxacin (21) Oral Cotrimoxazole (21)	None	Death (unrelated to OM)
4	Tarsal and Metatarsals	Unable to discern	IV Cloxacillin (3) IV Tazocin (7) IV Vancomycin (4)	Oral Cloxacillin (42) Oral Fluconazole (15)	None	Palliative care for underlying osteosarcoma
5	Proximal Tibia	Unable to discern	N.A.	IV Clindamycin (43) Oral Cotrimoxazole (74)	Incision and drainage/ Curettage (1)	Death (unrelated to OM)
6	Humerus	Unable to discern	N.A.	IV Clindamycin (42)	Incision and drainage/ Curettage (1)	Death (unrelated to OM)
7	Proximal Tibia	Methicillin-resistant *Staphylococcus aureus*	N.A.	IV Vancomycin (14) IV Clindamycin (30) Oral Clindamycin (14)	Incision and drainage/ Curettage (1)	*Complete resolution
8	Distal Femur	Methicillin-susceptible *Staphylococcus aureus*	N.A.	IV Cloxacillin (27) Oral Rifampicin (39) Oral Cefalexin (14)	Incision and drainage/ Curettage (1)	*Complete resolution
9	Phalanx of Hand	*Mycobacterium tuberculosis*	IV Co-amoxiclav (1) IV Cloxacillin (3)	Oral Rifampicin (365) Oral Isoniazid (365) Oral Pyrazinamide (60) Oral Ethambutol (60)	None	Death (unrelated to OM)
10	Distal Femur	*Mycobacterium bovis* (BCG)	IV Cloxacillin (7)	Oral Cefalexin (35) Oral Rifampicin (158) Oral Ethambutol (158) Oral Levofloxacin (158)	Incision and drainage/ Curettage (1)	Poor bone growth
11	Base of Skull	*Rhizopus* species	IV Tazocin (30) IV Amikacin (12) IV Meropenem (4) IV Caspofungin (4) IV Voriconazole (9)	IV Amphotericin/ Ambisome (327) Oral Posaconazole (215)	Examination under Anaesthesia Functional Endoscopic Sinus Surgery Craniotomy and Debridement	Poor bone growth
12	Distal Femur	*Burkholderia pseudomallei*	IV Ceftriaxone (13) IV Metronidazole (1)	IV Co-amoxiclav (14) IV Ceftriaxone (14) Oral Co-amoxiclav (180) Oral Cotrimoxazole (180)	Incision and drainage/ Curettage (1)	Poor bone growth
13	Proximal Fibula	*Pseudomonas aeruginosa*	N.A.	IV Ciprofloxacin (6) Oral Ciprofloxacin (34) IV Ceftazidime (28)	Incision and drainage/ Curettage (1)	Poor bone growth
14	Talus	*Mycobacterium bovis* (BCG)	IV Cloxacillin (3)	IV Amikacin (16) Oral Rifampicin (252) Oral Isoniazid (252) Oral Pyrazinamide (25) Oral Ethambutol (252)	Incision and drainage/ Curettage (2)	Poor bone growth

*Based on clinical symptoms and normal C-reactive protein values

Atypical pathogens were isolated in eight patients (57.1%). There were three cases with Gram negative bacteria isolated (21.4%) which included *Stenotrophomonas maltophilia, Burkholderia pseudomallei* and *Pseudomonas aeruginosa.* Mycobacteria was isolated in three cases (21.4%) including two cases of *Mycobacterium bovis* and one case of *Mycobacterim tuberculosis.* One case had *Rhizopus* species isolated. *Staphylococcus aureus*, which is a common cause of OM, was only isolated in two patients (14%).

In light of our patients’ compromised immune status and/or their unusual causes of OM, prolonged courses of appropriate antimicrobials were given for a duration ranging from 6 weeks to 1 year. We also found extended periods of raised CRP in our patients, lasting up to 159 days despite extended antimicrobial courses. Surgery (incision and drainage/curettage) was performed in 11 patients (79%). Wherever possible, the patients received treatment for their underlying immunodeficiency. Only three patients (21%) recovered completely - resolution of OM without long term sequelae such as poor bone growth or chronic discharging sinuses. Five patients (36%) had poor bone growth (e.g. limb length discrepancy), while one patient had recurrent discharge from the bone. Four patients (29%) died from their underlying conditions unrelated to OM while one patient received palliative care for underlying osteosarcoma and was not followed up for OM. Our patients’ site of infection, pathogen isolated, treatment regimen, surgery and outcome are summarised in Table 3.

## Discussion

In developed countries, acute OM occurs in about 8 in 100,000 children, with boys being affected more than girls [15]. The classical clinical picture includes an unwell and pyrexic child with pain and signs of inflammation around a long bone. The most common sites include the femur (23–29%), tibia (19–26%) and humerus (5–13%) [5]. Most of our cases involved the long bone, which is congruent with the literature [5]. However, we have also demonstrated that unusual infection sites need to be considered in immunocompromised patients. We reported infections of the tarsal and metatarsal bones, phalanges of the hand and base of the skull. These are sites that are estimated to be involved in less than 1% of OM cases [5].

The classical presentation of a child with OM includes fever, bone pain, reduced ambulation and bone swelling. They can also present with generalised malaise. Severe tenderness, reduced range of movement, local oedema, erythema and warmth can be commonly found on physical examination [4, 5] Our case series highlights that immunocompromised children are less likely to present with these classical features which could be due to the inability of a compromised immune system to produce an adequate inflammatory response [13]. About 64% of our patients presented with a fever over 38 °C, 66.7% presented with bone pain and 28.6% presented with reduced active mobility. Their sensitivity in detecting OM in our patient pool are 64.3, 42.9 and 28.6% respectively. This demonstrates the difficulty of clinically diagnosing a immunocompromised child with OM. However, features like bone swelling and raised CRP which were positive in > 90% of our patients. This is in line with literature that CRP is a sensitive marker in detecting acute OM and a CRP of < 20 mg/L makes acute OM a less likely diagnosis [9, 12]. Our findings reaffirm that there should be a high index of clinical suspicion for OM in known immunocompromised pediatric patients. CRP should be measured on admission and early imaging obtained to facilitate early diagnosis and initiation of empirical treatment.

To avoid the development of sequelae, OM is usually treated aggressively and empirically with broad spectrum IV antimicrobials before the causative pathogen is cultured and identified [5, 12]. Studies have shown that a delay in the initiation of antimicrobials led to sepsis, lower resolution rate and an increased incidence of sequelae such as abscess formation or chronic OM [16, 17]. Once the sensitivities are established, treatment can be adjusted based on antimicrobial sensitivities. Commonly used empirical antimicrobials include anti-staphylococcal agents (cloxacillin, flucloxacillin), third generation cephalosporins (cefotaxime, ceftriaxone) and lincosamides (clindamycin) [12]. We treated all immunocompromised patients with appropriate empirical antimicrobials which included broad spectrum agents such as co-amoxiclav, piperacillin-tazobactam (Tazocin), meropenem, amikacin and third generation cephalosporins like ceftriaxone and ceftazidime. The initial use of broad spectrum empirical antimicrobials is important in the early treatment of OM in immuncompromised patients as there is an increased chance for isolating atypical pathogens.

The typical course of antimicrobials for acute OM is 20 days [14]. The first two to 4 days would be the IV phase. A switch to oral antimicrobials may be appropriate if the patient improves clinically and the CRP is normalising [9, 12, 14, 18]. This was not the case in our patients. We used longer courses of targeted antimicrobial treatment with extended IV phases to manage OM in immunocompromised patients and neonates. The length of the IV phase in our patients ranged from 3 to 327 days. Three of our patients had short IV phases that lasted 7 days or less. This was because they were found to be infected with either *Mycobacteria tuberculosis* or *Mycobacteria bovis* (BCG) and were switched on to appropriate prolonged courses of oral antimycobacterial regimens. This demonstrates that limited courses of IV antimicrobials is not sufficient to treat OM in immunocompromised children. The patient and their parents should be counselled for prolonged courses of IV antimicrobials as well as the need for adjuncts such as peripherally inserted central catheters (PICC).

As mentioned above, CRP is a sensitive marker for detecting OM. CRP is also useful as an indicator of response to antimicrobial treatment and clinical course of the disease. A clinically improving patient with a CRP of less than 20 mg/L has been shown to be an indication to stop antimicrobial therapy [12, 14]. Our study has shown that in immunocompromised children, the normalisation of CRP to less than 20 mg/L is delayed. There are extended periods of raised CRP, lasting up to 159 days despite prolonged antimicrobial courses. This may imply the necessity for longer antimicrobial regimes with an extended IV phase. This also demonstrates that CRP remains a good indicator of response to antimicrobial treatment and clinicians should take it into consideration when deciding on duration of antimicrobials or the need for further management such as surgery.

The indications for surgical intervention in the management of OM in children is complicated and controversial [5]. Commonly accepted indications are soft tissue abscess formation, bone sequestrum, concomitant septic arthritis or failure to respond to antibiotic treatment [19–21]. With advancements in antimicrobial treatment, the rates of surgical intervention have decreased and some studies have shown that antimicrobial therapy alone could be sufficient in 90% of cases of OM in immunocompetent patients [3, 14, 22]. Our study has demonstrated that unusual pathogens have been isolated in our immunocompromised patients such as Gram negative bacteria and fungi. We find that unlike immunocompetent patients, in immunocompromised patients there is a role for surgery to obtain samples for microbiology to allow for targeted antimicrobials as the empirical antimicrobials may not be appropriate to treat these atypical pathogens.

There is also limited information on the efficacy of a non-surgical approach in immunocompromised children with OM. In our study, only three patients did not undergo surgery. The rest of the patients had undergone at least one procedure involving drainage/curettage. Three patients had to undergo multiple procedures due to failure of their OM to resolve, as well as repeated abscess formation. Our immunocompromised patients have a reduced response to antimicrobial treatment as seen by the need for prolonged antimicrobial regimens and delay in clinical improvement. Mechanical debulking in the form of surgical debridement of the infected tissue could play a significant role in reducing the bacterial load to better combat the infection [19]. This implies that surgery plays an important role in managing OM in immunocompromised patients and multiple debridement may be required.

After treatment, resolution of OM without sequelae was seen in only 3 (21.4%) patients. Two neonates achieved immune maturity and one patient who had undergone a bone marrow transplant for Fanconi anemia achieved complete resolution of their OM. This shows that along with antimicrobial and surgical treatment, recovery of the immune system could contribute significantly to a higher chance of complete resolution without long term sequelae and treatment for underlying immunodeficiency should be attempted in patients where possible.

This is a case series with 14 patients. We hope to perform a further study with more patients in the future that will provide data with more statistical significance to further validate our findings.

## Conclusion

In conclusion, although uncommon, OM in immunocompromised children and neonates can be caused by unusual pathogens, and can infect unusual sites, with devastating effects. Treatment involves prolonged administration of antibiotics and surgery. In light of unusual causative pathogens, surgery also has an important role for collection of tissue samples for microbiological studies to allow for targeted antimicrobial therapy. CRP remains a useful marker in diagnosing and monitoring for improvement. However, CRP has been found to take longer to normalise in immunocompromised patients. Further affirming the need for prolonged IV antimicrobial treatment and possibly repeated surgical debridement. Immune recovery seems to play an important role in bone healing and recovery, and treatment for immunodeficiency should be attempted where possible.

## Data Availability

The datasets generated during this study are not publicly available to preserve patient confidentiality but are available from the corresponding author on reasonable request.

## Abbreviations

OM: Osteomyelitis
CRP: C-reactive protein
MRI: Magnetic Resonance Imaging
MSMD: Mendelian susceptibility to mycobacterial disease
HLH: Familial haemophagocytic lymphohistiocytosis
